# AIP augments CARMA1-BCL10-MALT1 complex formation to facilitate NF-κB signaling upon T cell activation

**DOI:** 10.1186/s12964-014-0049-7

**Published:** 2014-07-22

**Authors:** Gisela Schimmack, Andrea C Eitelhuber, Michelle Vincendeau, Katrin Demski, Hisaaki Shinohara, Tomohiro Kurosaki, Daniel Krappmann

**Affiliations:** 1 Research Unit Cellular Signal Integration, Institute of Molecular Toxicology and Pharmacology, Helmholtz Zentrum München-German Research Center for Environmental Health, Ingolstädter Landstr. 1, Neuherberg, 85764, Germany; 2 Laboratory for Lymphocyte Differentiation, RIKEN Research Center for Integrative Medical Sciences (IMS), 1-7-22, Suehiro-cho, Tsurumi-ku, Yokohama 230-0045, Kanagawa, Japan; 3 Laboratory of Lymphocyte Differentiation, WPI Immunology Frontier Research Center, Osaka University, 3-1 Yamadaoka, Suita 565-0871, Osaka, Japan

**Keywords:** Immunology, T cell signaling, Canonical NF-κB, MAGUK family

## Abstract

**Background:**

The CARMA1-BCL10-MALT1 (CBM) complex bridges T cell receptor (TCR) signaling to the canonical IκB kinase (IKK)/NF-κB pathway. The CBM complex constitutes a signaling cluster of more than 1 Mio Dalton. Little is known about factors that facilitate the rapid assembly and maintenance of this dynamic higher order complex.

**Findings:**

Here, we report the novel interaction of the aryl hydrocarbon receptor (AHR) interacting protein (AIP) and the molecular scaffold protein CARMA1. In T cells, transient binding of CARMA1 and AIP enhanced formation of the CBM complex. Thereby, AIP promoted optimal IKK/NF-κB signaling and IL-2 production in response to TCR/CD28 co-stimulation.

**Conclusions:**

Our data demonstrate that AIP acts as a positive regulator of NF-κB signaling upon T cell activation.

## Findings

Assembly of the CARMA1-BCL10-MALT1 (CBM) complex is an essential step in the signal transmission from the T cell receptor (TCR) to the activation of canonical IκB kinase (IKK)/NF-κB signaling [[1]]. After TCR/CD28 co-stimulation, receptor-proximal signaling events at the immunological synapse lead to activation of protein kinase C θ (PKCθ), which in turn phosphorylates the scaffold protein CARMA1. Hereby a conformational change of CARMA1 is induced that enables the recruitment of pre-assembled BCL10-MALT1 complexes [[2],[3]]. This process is accompanied by the association of many other factors to the CBM complex such as TRAF6, Caspase8, CK1α, CSN5, A20 and PP2A that control CBM activity and downstream signaling [[4]]. CARMA1 belongs to the family of MAGUK (membrane-associated guanylate kinase) proteins comprising PDZ, SH3 and GUK domains in its C-terminus. CARMA1 expression is restricted to lymphoid cells where it is associated to the cytosolic membrane.

**Figure 1:**
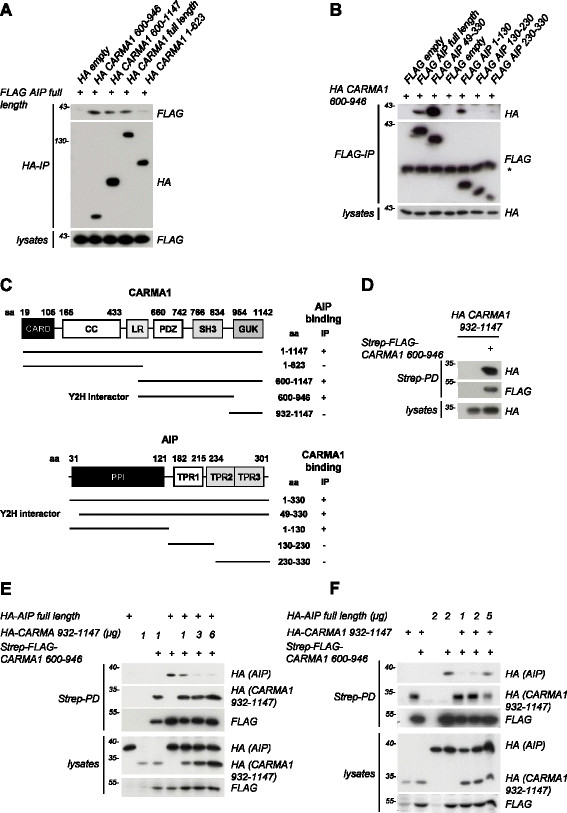
**AIP interacts with CARMA1 in overexpression experiments and thereby competes with intramolecular CARMA1 association. (A)** Interaction of overexpressed CARMA1 aa 600–946 (PDZ-SH3), CARMA1 aa 600–1147 (C-term) and CARMA1 full length with AIP full length, while CARMA1 aa 1–623 (N-term) is not interacting with AIP. HEK293 cells were co-transfected with FLAG-AIP and different HA-tagged CARMA1 constructs as indicated. After lysis, co-immunoprecipitation (co-IP) was carried out using anti-HA antibody and analysed by Western Blotting. **(B)** Interaction of overexpressed AIP full length, AIP aa 49–330 (Y2H interactor) and AIP aa 1–130 (PPI domain) with CARMA1 aa 600–946. AIP aa 130–230 (TPR1) and AIP aa 230–330 (TPR2 and 3) are not interacting. Experiment was performed analogous to **(A)** as anti-FLAG IP. Asterisk indicates migration of IgGs. **(C)** Schematic summary of the interaction between CARMA1 and AIP fragments. **(D)** Interaction of HA-CARMA1 aa 932–1147 (GUK) with Strep-FLAG-CARMA1 aa 600–946 (PDZ-SH3). HEK293 cells were co-transfected with both CARMA1 constructs and the binding was analyzed by Western Blotting after Strep-Tactin PD. **(E, F)** AIP and CARMA1 aa 932–1147 (GUK) bind to CARMA1 aa 600–946 (PDZ-SH3) in a competitive manner. **(E)** HEK293 cells were co-transfected with Strep-FLAG-CARMA1 aa 600–946, HA-AIP and rising concentrations of HA-CARMA1 aa 932–1147. Strep-Tactin PD was performed as in **(D)**. **(F)** HEK293 cells were co-transfected and analyzed essentially as in **(E)**, however using constant amounts of HA-CARMA1 aa 932–1148 and rising concentrations of HA-AIP.

To identify novel CARMA1 interaction partners, we had previously performed yeast-two-hybrid (Y2H) screens using C-terminal CARMA1 constructs as baits [[5]]. Besides the protein phosphatase PP2A, we identified the aryl hydrocarbon receptor (AHR) interacting protein (AIP) as a new interaction partner of the C-terminal PDZ-SH3 domain of CARMA1 in yeast (data not shown). Using co-immunoprecipitation (co-IP) after transfection of HEK293 cells, we confirmed the association of CARMA1 and AIP (Figure 1A–C; see Additional file 1 for detailed Methods description). Full length HA-CARMA1 and FLAG-AIP interacted after anti-HA or anti-FLAG IP, respectively. In agreement with the data from the Y2H screen, AIP bound to C-terminal CARMA1 fragments that contain the PDZ-SH3 domains, but not to the CARMA1 N-terminus (Figure 1A). Vice versa mapping of the CARMA1 interaction surface on AIP revealed that the N-terminal peptidyl-prolyl *cis/trans* isomerase (PPI) domain is binding to the CARMA1 PDZ-SH3, while the C-terminal tetratricopeptide repeats (TPRs) are dispensable (Figure 1B).

Initially, AIP has been proposed to regulate the aryl hydrocarbon receptor (AHR) localization, stability and ligand receptivity [[6],[7]]. AIP was shown to bind Hsp90 and AHR primarily through the TPR and suggested to act in complex with Hsp90 to regulate ligand-triggered AHR responses [[8]-[10]]. The N-terminal FKBP-like PPI domain of AIP that we identified as the CARMA1 interaction surface does not confer enzymatic activity [[11]-[13]]. Since extensive intramolecular restructuring of the CARMA1 MAGUK region is required to initiate downstream signaling after T cell stimulation [[14]], we asked if AIP can support conformational changes involved in CBM formation. For MAGUK proteins like DLG (discs large) and PSD95 an inhibitory intramolecular association between the SH3 domain and the C-terminal GUK domain has been demonstrated [[15],[16]]. Strep-Tactin pull-down (PD) revealed that the CARMA1 GUK domain directly binds to the PDZ-SH3 domain in HEK293 cells (Figure 1D). Since no direct association between AIP and CARMA1 GUK was obtained, we asked whether the CARMA1 GUK and AIP may compete for the same binding surface on CARMA1 and co-expressed HA-GUK and HA-AIP together with Strep-FLAG-PDZ-SH3 (Figure 1E and F). Again, CARMA1-GUK or AIP alone interacted with CARMA1-PDZ-SH3. Upon co-transfection, increasing concentrations of HA-GUK or HA-AIP led to a dose dependent loss of PDZ-SH3 association to AIP or CARMA1 GUK, respectively. Thus, AIP and CARMA1 GUK compete for the same surface on CARMA1, suggesting that their binding is mutually exclusive. In this setting, the CARMA1 GUK seemed to have a higher affinity for PDZ-SH3, which could keep CARMA1 in an inactive state. However, AIP binding may either facilitate an opening of this intramolecular interaction or stabilize the open conformation to alleviate CARMA1 activation and downstream signaling.

**Figure 2:**
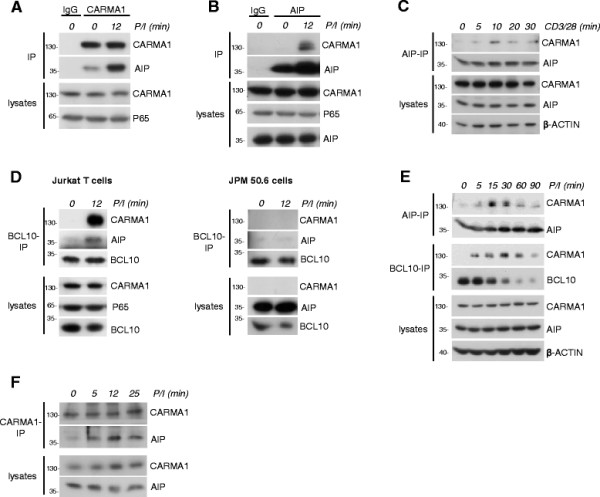
**AIP interacts with CARMA1 after T cell stimulation. (A, B)** Interaction of CARMA1 and AIP in Jurkat T cells after P/I stimulation. **(A)** Cells were left untreated or P/I treated as indicated prior to anti-CARMA1 or IgG control IP, respectively. Co-IP was analyzed by Western Blotting. **(B)** Jurkat T cells were treated as in **(A)** and after lysis subjected to anti-AIP or IgG control IP. **(C)** Transient interaction of CARMA1 and AIP after CD3/CD28 co-stimulation. Jurkat T cells were stimulated as indicated prior to anti-AIP IP and analyzed by Western Blotting. **(D)** AIP – CBM complex association requires CARMA1. Jurkat T cells or CARMA1 deficient JPM50.6 T cells were stimulated with P/I as indicated and subjected to anti-BCL10 IP. Western Blots were stained for AIP co-precipitation. **(E)** AIP and BCL10 show similar binding kinetics to CARMA1 after T cell stimulation. Jurkat T cells were in parallel stimulated with P/I as indicated and after lysis subjected to anti-AIP or BCL10 IP, respectively. Western Blots were stained for CARMA1 co-precipitation. **(F)** Transient interaction of CARMA1 and AIP in primary mouse CD4 T cells. Cells were left untreated or stimulated with P/I as indicated prior to anti-CARMA1 IP. Co-IP was analyzed by Western Blotting.

To investigate if the CARMA1-AIP association is relevant for T cell signaling, we first confirmed an endogenous interaction in Jurkat T cells (Figure 2). Co-IPs using anti-CARMA1 or anti-AIP antibodies showed a transient interaction in the initial phase of T cell stimulation after PMA/Ionomycin (P/I) treatment or by CD3/CD28 co-ligation (Figure 2A-C). CARMA1 recruits BCL10-MALT1 to assemble the CBM complex and we asked if AIP is associated with the CBM holo-complex by performing anti-BCL10 IPs (Figure 2D). Indeed, AIP is also precipitated with BCL10 after T cell stimulation and this interaction was not seen in CARMA1 deficient JPM50.6 Jurkat T cells [[17]], revealing that the AIP - CBM association is mediated through CARMA1. To obtain further evidence that AIP could be involved in CBM regulation, we performed parallel time course analyses of AIP-CARMA1 and BCL10-CARMA1 association after P/I stimulation of Jurkat T cells (Figure 2E). CARMA1 co-precipitated with AIP and BCL10 at early time points after T cell stimulation. CARMA1-AIP binding was lost after pro-longed treatment, when the CBM complex was destroyed due to BCL10 degradation [[18],[19]]. Next we asked if the CARMA1-AIP association is also relevant in primary T cells. AIP is expressed in primary human as well as mouse T cells as detected by Western Blotting (Additional file 2A-B) and AIP mRNA levels did not significantly change in response to CD3/CD28 stimulation (Additional file 2C). By co-IPs we could verify a stimulus dependent interaction of CARMA1 and AIP in murine CD4 T cells (Figure 2F). Thus, just like the competition experiments of AIP and CARMA1 GUK for CARMA1 PDZ-SH3, the endogenous association studies suggest that AIP is predominantly bound to the open and active CARMA1 conformation within the CBM complex.

**Figure 3:**
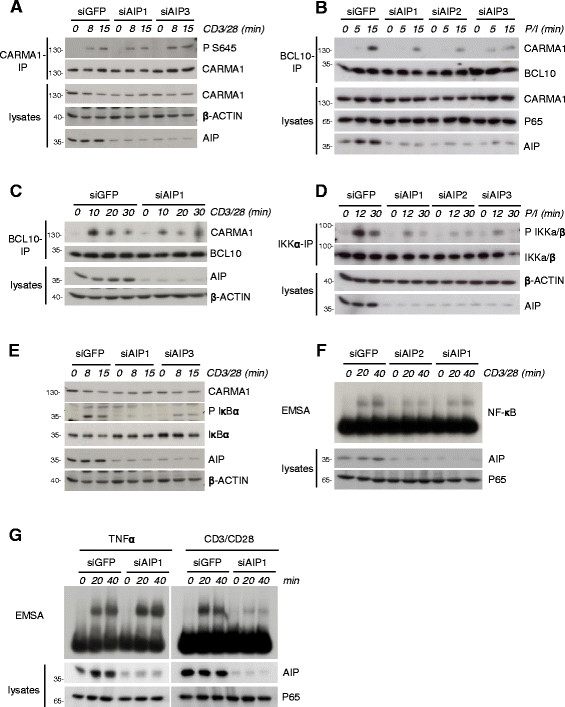
**AIP positively regulates CBM complex formation and NF-κB activity in Jurkat T cells. (A)** AIP knockdown does not affect CARMA1 Ser645 phosphorylation. Jurkat T cells were transfected with GFP or AIP targeting siRNAs 1 and 3 and stimulated with CD3/CD28 as indicated. After anti-CARMA1 IP, CARMA1 phosphorylation was detected by a phospho-Ser645-specific antibody. **(B, C)** AIP is required for CARMA1-BCL10 association. Jurkat T cells were transfected with siRNAs targeting GFP or AIP (siAIP1, 2, 3) and stimulated with P/I **(B)** or siAIP1 and 3 and stimulated with CD3/CD28 **(C)** as indicated. Cells were lysed and subjected to anti-BCL10 IP. **(D)** AIP knockdown impairs IKK T-loop phosphorylation. Jurkat T cells were transfected with siRNAs as in **(B)** and after P/I stimulation subjected to anti-IKKα IP. After Western Blotting, T-loop phosphorylation was detected with a phospho-specific antibody. **(E)** IκBα phosphorylation and degradation is reduced after AIP knockdown. Jurkat T cells were transfected with siRNAs as in **(A)** and stimulated by CD3/CD28 co-ligation as indicated. IκBα phosphorylation and degradation was analyzed by western blotting. **(F)** AIP knockdown diminishes NF-κB activation. Jurkat T cells were transfected with siRNA against GFP or siAIP1 and 2 and stimulated with anti-CD3/CD28 antibodies as indicated. NF-κB DNA binding was assessed by EMSA. **(G)** AIP does not influence TNFα induced NF-κB activity. Jurkat T cells were transfected with siRNAs against GFP and AIP (siAIP1), respectively, and stimulated with TNFα or CD3/CD28 for the indicated time points. NF-κB DNA binding was assessed by EMSA.

To address if AIP is involved in CBM complex formation and downstream signaling in Jurkat T cells, we performed knockdown experiments of AIP using a panel of three independent siRNAs (siAIP1, siAIP2 and siAIP3). A crucial step to initiate CBM assembly represents PKC dependent phosphorylation at Ser645 within the linker region of CARMA1 [[3],[5]]. We downregulated AIP and detected Ser645 phosphorylated CARMA1 by a phospho-specific antibody (Figure 3A) [[20]]. There was no difference in the extent of CARMA1 S645 phosphorylation upon CD3/CD28 stimulation, implying that AIP did not impair initial PKC-mediated CARMA1 linker phosphorylation. To see if AIP knockdown has an influence on CBM complex formation, we directly determined CARMA1 association after anti-BCL10 co-IP in response to P/I or CD3/CD28 stimulation (Figure 3B and C). In all cases there was a significant decrease in CARMA1-BCL10 association after AIP knockdown, demonstrating the necessity of AIP for proper CBM complex formation after T cell stimulation.

CBM complex formation is the key step for activation of canonical IKK/NF-κB signaling in response to TCR/CD28 co-engagement [[1]]. To assess if AIP also affects canonical NF-κB signal transmission downstream of the CBM complex, we determined IKK activation (Figure 3D), IκBα phosphorylation and degradation (Figure 3E) and NF-κB activation (Figure 3F) in AIP knockdown cells using different siRNAs. Clearly, IKKα/β T loop phosphorylation and thus IKK activity was severely reduced in AIP depleted Jurkat T cells. Further, less IKK activation was accompanied by reduced IκBα phosphorylation and degradation and decreased nuclear NF-κB DNA binding in response to P/I or CD3/CD28 stimulation. To address if AIP controls selectively TCR signaling, we compared NF-κB activation after CD3/CD28 and TNFα stimulation in AIP knockdown cells. Indeed, reduction of AIP selectively diminished NF-κB activity after CD3/CD28, but not after TNFα stimulation (Figure 3G), suggesting that AIP is modulating IKK/NF-κB upstream of IKK by regulating CBM-complex formation. Further, we determined the role of AIP for activation of the MAPKinases ERK and JNK in response to P/I stimulation (Additional file 3). Whereas ERK phosphorylation was largely independent of AIP, phosphorylation of JNKp54 isoform was slightly decreased, which is in line with previous findings that CARMA1-BCL10 are involved in JNKp54 activation [[21]].

**Figure 4:**
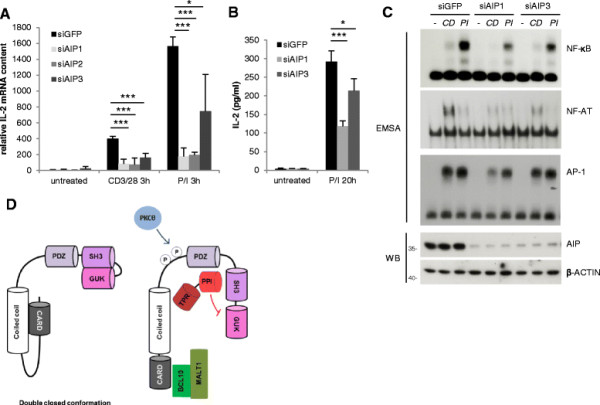
**AIP regulates optimal T cell signaling responses. (A)** IL-2 mRNA levels are decreased in AIP knockdown cells. Jurkat T cells were transfected with siGFP or siAIP1 and 3 and stimulated with P/I or CD3/CD28 for 3 h. RNA was isolated and IL-2 transcript levels were analyzed by quantitative RT-PCR. Bars show average and standard deviation of three independent experiments. **(B)** Downregulation of AIP reduces IL-2 production. Jurkat T cells were transfected with siRNAs (siAIP1, 2 and 3) as in **(A)** and stimulated with P/I. Secreted IL-2 was measured by ELISA. In A and B bars show average and standard deviation of three independent experiments. Significance was evaluated using Student *t*-test (one star: p < 0,05; three stars p < 0,001). **(C)** AIP knockdown diminishes NF-κB and NF-AT activation, while AP-1 activation is only slightly affected. Jurkat T cells were transfected with siRNAs against GFP and AIP (siAIP1 and 3), respectively, and stimulated with P/I or CD3/CD28 for 3 hours. NF-κB, NF-AT and AP-1 DNA binding was assessed by EMSA. **(D)** Working model how AIP affects CARMA1. Upon CARMA1 linker phosphorylation by PKCθ, AIP binding to PDZ-SH3 can compete for intramolecular GUK-SH3 interaction, which may facilitate structural rearrangements to transfer the double-closed conformation of CARMA1 into an active open state.

To assess the downstream consequences of diminished TCR signaling, we determined interleukin-2 (IL-2) production as a hallmark of T cell activation after siRNA mediated AIP downregulation. As measured by quantitative RT-PCR, upregulation of *IL-2* mRNA in response to T cell stimulation by P/I or CD3/CD28 stimulation was significantly impaired by AIP knockdown (Figure 4A). Congruently, P/I induced IL-2 production and secretion was also decreased in AIP depleted Jurkat T cells as determined by ELISA (Figure 4B). IL-2 induction in T cells does not only rely on IKK/NF-κB signaling, but also on activation of the transcription factors NF-AT and AP-1 [[22]]. Therefore, we also assessed NF-AT and AP-1 DNA binding in nuclear extracts of Jurkat T cells after AIP knockdown (Figure 4C). NF-AT was severely reduced and AP-1 was slightly diminished in AIP downregulated Jurkat T cells, highlighting that AIP augments TCR/CD28 downstream pathways that contribute to optimal IL-2 induction. Effects on AP-1 may be downstream of NF-κB, because NF-κB activation can contribute to expression of Jun/AP-1 transcription factors such as JUNB and JUND [[23]]. Intriguingly, the MAGUK family member DLGH1 was shown to be an essential factor for TCR-triggered NF-AT activation, which may indicate that AIP could be a general regulatory factor for MAGUK dependent signaling events [[24]].

Taken together, our data reveal a novel and unexpected role of AIP as a positive regulator of CBM complex formation and canonical IKK/NF-κB as well as NF-AT signaling in activated T cells. The observed effects of AIP are independent of AHR, as we and others could not detect AHR expression in Jurkat T cells [[25]]. In its inactive state, CARMA1 was suggested to adopt a double-closed conformation with the N-terminal CARD bound to the coiled-coil and the C-terminal GUK associated to the SH3 [[14]] (scheme Figure 4D). Whereas BCL10-MALT1 association to the CARD opens up the N-terminus of CARMA1, AIP interacts with the PDZ-SH3 and may thereby facilitate loss of C-terminal GUK-SH3 interaction to support opening of the C-terminal MAGUK region and downstream signaling. In this model, AIP binding may support the opening of CARMA1 or stabilize the signal competent active conformation. Since CARMA1 conformation is regulated by a multistep process, AIP may also function in signal amplification and/or positive feedback loop [[26]]. Recent data reveal that within the CBM complex CARMA1 acts as a molecular seed that initiates the assembly of BCL10 filamentous fibers [[27]]. It is tempting to speculate that AIP as a cofactor may guide the complex assemblies of such higher order molecular clusters that are initiated by rearrangements of MAGUK family members.

## Abbreviations

AHR: Aryl hydrocarbon receptor

AIP: AHR interacting protein

CARD: Caspase recruitment domain

CC: Coiled-coil

CBM: CARMA1-BCL10-MALT1

GUK: Guanylate kinase

IKK: IκB kinase

IL-2: Interleukin 2

IP: Immunoprecipitation

LR: Linker region

MAGUK: Membrane-associated guanylate kinase

NF-AT: Nuclear factor of activated T-cells

NF-κB: Nuclear factor-κB

P/I: PMA/Ionomycin

PDZ: PSD95, DLG1, ZO1

PKCθ: Protein kinase C θ

PMA: Phorbol 12-myristate 13-acetate

PPI: Peptidyl-prolyl *cis/trans* isomerase

TCR: T-Cell receptor

TPR: Tetratricopeptide repeat

SH3: SRC homology 3

## Competing interests

The authors declare that they have no competing interests.

## Authors’ contributions

GS designed and conducted most experiments, analyzed the data and wrote the manuscript. ACE, MV and KD designed and conducted experiments and analyzed the data. SH and TK generated and contributed important material. DK conceived the project, analyzed the data and wrote the manuscript. All authors read and approved the final version of the manuscript.

## Additional files

## Supplementary Material

Additional file 1: Methods description. Click here for file

Additional file 2: AIP is expressed in primary mouse and human T cells. Click here for file

Additional file 3: Effects of AIP on MAP kinase activation. Click here for file
