# Supplementary material for: AIP augments CARMA1-BCL10-MALT1 complex formation to facilitate NF-κB signaling upon T cell activation
Source: Cell Commun Signal. 2014 Jul 22;12:49. doi: 10.1186/s12964-014-0049-7 (PMC4222456; doi:10.1186/s12964-014-0049-7)
Supplement: Additional file 1: — Methods description. [file s12964-014-0049-7-S1.docx]

**ADDITIONAL FILE 1 SCHIMMACK ET AL.**

**Methods description**

Antibodies, reagents, siRNAs and plasmids

The following antibodies were used: human CD3, human CD28, mouse IgG1 and mouse IgG2a, IKKα (556532 (IP)) (all BD Biosciences); p-Tyrosin (4G10; Millipore); ERK1/2 (442704, Calbiochem); BCL10 (331.3 (WB) C17 (IP)), β-ACTIN (I-19), CARMA1 (N20 (IP)), P65 (A), IKKα/β (H470 (WB)) (all Santa Cruz Biotechnology); AIP (35-2) (Biozol); CARMA1 (1D12 (WB)), p-ERK1/2 (9101), IκBα (L35A5), p-IκBα (5A5), phospho-IKKα/β, JNK1/2 (9252), p-JNK1/2 (9255) (all Cell Signaling); Phospho-specific antibody against chicken Ser668/human Ser645 CARMA1 [[1](#_ENREF_1)]; FLAG-M2 (Sigma); HA (clone 3F1; obtained from E. Kremmer). The following reagents and short interfering (si) RNAs (100 nM) were used: Phorbol 12-myristate 13-acetate (PMA), Ionomycin (Calbiochem); recombinant human TNFα (Biomol); Protease inhibitor cocktail (Roche); Protein G Sepharose (GE Healthcare); Strep-Tactin Sepharose (IBA Lifesciences); siGFP control, siAIP1: GCAACATCGCGGTGGGCAA, siAIP2: AGAAGGACGAAGAGGACAA (all Eurogentec); si AIP3 (smart pool of 4 selective siRNAs; Thermo Scientific); all tagged CARMA1 and AIP cDNAs were cloned in the pEF backbone vector (Invitrogen).

Cell culture

Standard cell culture was performed as described previously [[2](#_ENREF_2)]. For P/I stimulation of Jurkat T cells and JPM50.6 cells, 200 ng/ml PMA and 300 ng/ml Ionomycin were applied. For CD3/CD28 co-ligation, 0.3 µg CD3 and 1 µg CD28 antibody was used in the presence of 0.5 µg rat anti-mouse IgG1 and IgG2a. For TNFα stimulation of Jurkat T cells 30ng/ml recombinant human TNFα was used; For RNA interference, Jurkat T cells were transfected with 100 nM siRNA and Atufect reagent (1,0 µg/ml) (Silence Therapeutics, Berlin) and analyzed after 72h. HEK293 cells were transfected using standard calcium phosphate precipitation protocols.

Purification of primary human T cells

To create the buffy coat, 16 U/ml heparine was added to human blood and centrifuged (300xg, RT, 10 min). The intermediate cell layer was collected and further purification was performed according to the Invitrogen protocol (Dynabeads Untouched Human T Cell Kit). Informed consent was obtained from donors of human blood.

Purification and Expansion of primary mouse T cells

Isolation of murine CD4 T cells from lymph nodes and spleen was performed according to the manufacturer´s protocol (Dynabeads 114.45; Invitrogen). Murine T cells were cultured in RPMI medium containing 10% FCS, 100 U/ml Penicillin/Streptomycin, 2 mM Glutamin and 50 µM β-Mercaptoethanol. Expansion was induced by stimulation on plate (0,5 µg/ml CD3, 1 µg/ml CD28) for two days and subsequent adding of 20 U/ml murine IL-2.

Co-Immunoprecipitation

For co-IP studies, Jurkat T cells, JPM50.6 cells or primary mouse T cells, respectively, were lysed in 900 µl co-IP buffer (25 mM HEPES pH 7.5, 150 mM NaCl, 0.2% NP-40, 10% glycerol, 1mM DTT, 10 mM sodium fluoride, 8 mM β-glycerophosphate, 300 µM sodium vanadate and protease inhibitor cocktail). For co-IPs in HEK293 cells a Triton X-100 containing co-IP buffer (50 mM Tris-HCl pH 7.4, 150 mM NaCl, 1 mM EDTA, 1% Triton X-100 and protease inhibitor cocktail) was used. IPs were performed by adding antibodies against AIP, BCL10, CARMA1, IKKα, FLAG-M2 or HA. Antibodies were incubated over night at 4°C. The next day Protein G Sepharose was added for 1-2h at 4°C. Afterwards, IPs were washed three times in lysis buffer without protease inhibitors. For Strep-Tactin Pulldowns in HEK293 cells, a lysis buffer containing 30mM Tris-HCl pH 7.4, 150 mM NaCl, 0.5% NP-40 and protease inhibitor cocktail was used. Strep-Tactin Pulldowns were performed using Strep-Tactin Sepharose (IBA Lifesciences).

Quantitative RT-PCR

Equal amounts of RNA (Qiagen RNeasy mini Kit) were DNase I treated with RQ1 RNAse free DNase (Promega) to generate DNA-free RNA. Subsequent LightCycler real-time PCR analysis was performed using SensiFAST SYBR No-ROX One-Step Kit (Bioline) according to the manufacturer’s protocol on a Roche LightCycler 480 (LC-480). Relative quantification of IL-2 transcripts was carried out with housekeeping genes as calibrators for normalization [[3](#_ENREF_3)]. The relative expression ratio was calculated from the real-time PCR efficiencies and the crossing point deviations of the target gene (IL-2) versus the housekeeping gene RNA polymerase II (RPII), as previously described [[4](#_ENREF_4)]. Primers: IL-2 forward primer 5’-CACAGCTACAACTGGAGCATTTAC-3’; IL-2 reverse primer 5’-TGCTGATTAAGTCCCTGGGTC-3’; RPII forward primer 5’-GCACCACGTCCAATGACA-3’; RPII reverse primer 5’- GTGCGGCTGCTTCCATAA-3’. For analysis of human and murine AIP transcripts, reverse transcription of DNAse treated mRNA was performed with total RNA using Superscript II (Roche Diagnostics, Mannheim) according to the manufacturer’s protocol. qRT-PCR was performed with the Roche LightCycler 480, using LightCycler FastStart DNA Master SYBR Green I-Kit and standard LightCycler protocol (Roche Diagnostics, Mannheim). The following primers were used for amplification: human AIP forward primer 5’- GACGAAGAGAAGGCAAAGGCAGTG-3’; human AIP reverse primer 5’- CTTCATCTGCAGGTTCTTGAGGCAGGC -3’; murine AIP forward primer 5’- GATGAGGAGAAAGCAAAGGCAGTA -3’; murine AIP reverse primer 5’- CTTCATCTGCAGATTCTTGAGGCAGGC -3’. RNA-Polymerase II served as internal standard, using primers given in [[3](#_ENREF_3)].

Electrophoretic Mobility Shift Assay (EMSA)

Nuclear extracts of stimulated cells were generated by lysis in buffer containing 10 mM

HEPES, 0.1 mM EDTA, 10 mM KCl, 1 mM dithiothreitol, 0.2% NP-40 and protease and

phosphatase inhibitors. After centrifugation, the supernatant contained cytoplasmic fraction while the pellet was further lysed in 20 mM HEPES, 10 % Glycerol, 0.4 M NaCl, 1 mM EGTA, 1 mM EDTA, 1 mM dithiothreitol plus protease and phosphatase inhibitors. After centrifugation, the supernatant contained the nuclear fraction. NF-κB and NF-AT EMSA were performed as previously described [[5](#_ENREF_5)]. AP-1 EMSA was performed analogous to NF-κB EMSA, using an oligonucleotide (GATCAGCTAGCATGAGTCAGACAC) comprising the TPA DNA response element (TRE).

Enzyme-linked ImmunoSorbent Assay (ELISA)

For IL-2 ELISA, cell culture supernatant from Jurkat T cells stimulated with P/I for 20 h was collected and secreted IL-2 was measured with human IL-2 ELISA kit (eBioscience) according to manufacturer’s protocol.

Additional References

1. Shinohara H, Maeda S, Watarai H, Kurosaki T: **IkappaB kinase beta-induced phosphorylation of CARMA1 contributes to CARMA1 Bcl10 MALT1 complex formation in B cells.** *The Journal of experimental medicine* 2007, **204:**3285-3293.

2. Scharschmidt E, Wegener E, Heissmeyer V, Rao A, Krappmann D: **Degradation of Bcl10 induced by T-cell activation negatively regulates NF-kappa B signaling.** *Molecular and cellular biology* 2004, **24:**3860-3873.

3. Radonic A, Thulke S, Mackay IM, Landt O, Siegert W, Nitsche A: **Guideline to reference gene selection for quantitative real-time PCR.** *Biochemical and biophysical research communications* 2004, **313:**856-862.

4. Pfaffl MW: **A new mathematical model for relative quantification in real-time RT-PCR.** *Nucleic acids research* 2001, **29:**e45.

5. Eitelhuber AC, Warth S, Schimmack G, Duwel M, Hadian K, Demski K, Beisker W, Shinohara H, Kurosaki T, Heissmeyer V, Krappmann D: **Dephosphorylation of Carma1 by PP2A negatively regulates T-cell activation.** *The EMBO journal* 2011, **30:**594-605.
