# Supplementary material for: AIP augments CARMA1-BCL10-MALT1 complex formation to facilitate NF-κB signaling upon T cell activation
Source: Cell Commun Signal. 2014 Jul 22;12:49. doi: 10.1186/s12964-014-0049-7 (PMC4222456; doi:10.1186/s12964-014-0049-7)
Supplement: Additional file 2: — AIP is expressed in primary mouse and human T cells. [file s12964-014-0049-7-S2.pptx]

## Slide 1
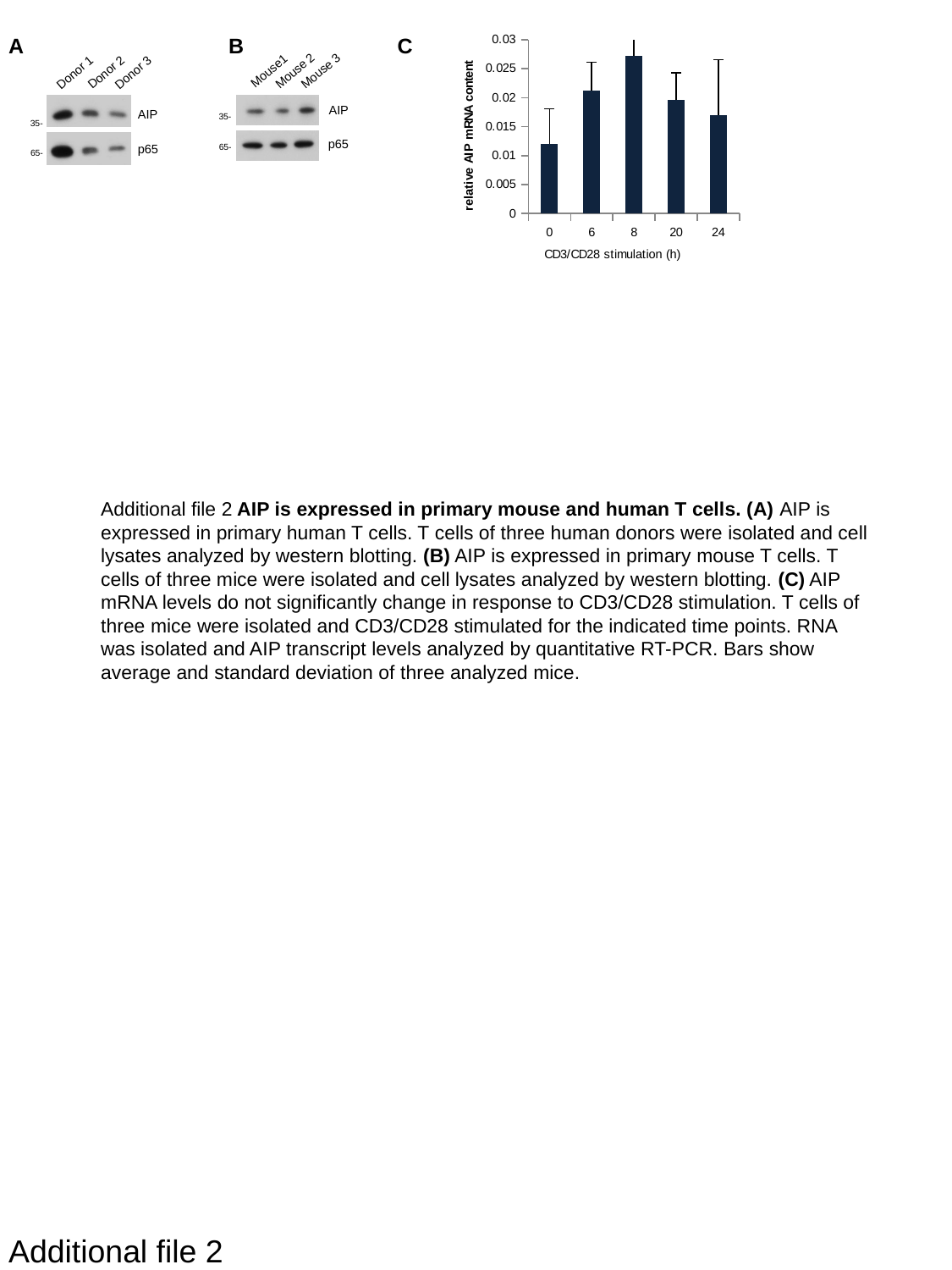

B
C
A
### Chart
| Category | |
|---|---|
| 0 | 0.012053491000000001 |
| 6 | 0.021259526666666674 |
| 8 | 0.02725004333333334 |
| 20 | 0.019540666666666668 |
| 24 | 0.01702080333333334 |Mouse 2
Mouse 3
Mouse1
AIP
p65
Donor 2
Donor 1
Donor 3
AIP
p65
35-
35-
65-
65-
Additional file 2 AIP is expressed in primary mouse and human T cells. (A) AIP is expressed in primary human T cells. T cells of three human donors were isolated and cell lysates analyzed by western blotting. (B) AIP is expressed in primary mouse T cells. T cells of three mice were isolated and cell lysates analyzed by western blotting. (C) AIP mRNA levels do not significantly change in response to CD3/CD28 stimulation. T cells of three mice were isolated and CD3/CD28 stimulated for the indicated time points. RNA was isolated and AIP transcript levels analyzed by quantitative RT-PCR. Bars show average and standard deviation of three analyzed mice.
Additional file 2
