# Supplementary material for: AIP augments CARMA1-BCL10-MALT1 complex formation to facilitate NF-κB signaling upon T cell activation
Source: Cell Commun Signal. 2014 Jul 22;12:49. doi: 10.1186/s12964-014-0049-7 (PMC4222456; doi:10.1186/s12964-014-0049-7)
Supplement: Additional file 3: — Effects of AIP on MAP kinase activation. [file s12964-014-0049-7-S3.pptx]

## Slide 1
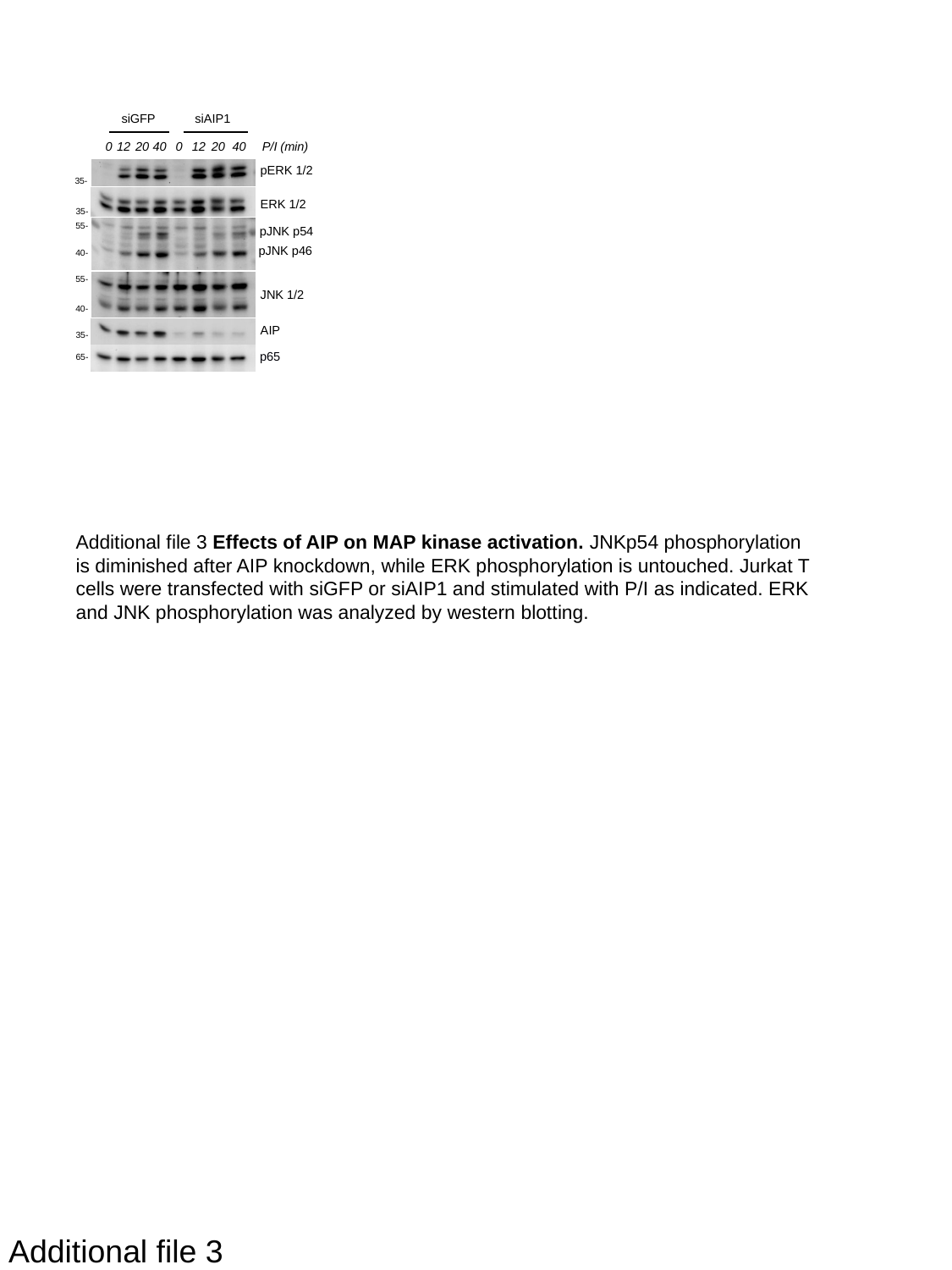

siGFP
siAIP1
0
12
20
40
0
12
20
40
P/I (min)
pERK 1/2
35-
ERK 1/2
35-
55-
pJNK p54
pJNK p46
40-
55-
JNK 1/2
40-
AIP
35-
p65
65-
Additional file 3 Effects of AIP on MAP kinase activation. JNKp54 phosphorylation is diminished after AIP knockdown, while ERK phosphorylation is untouched. Jurkat T cells were transfected with siGFP or siAIP1 and stimulated with P/I as indicated. ERK and JNK phosphorylation was analyzed by western blotting.
Additional file 3
